# Influence of Pyrolysis Temperature on the Properties and Electrochemical Performance of Cedar Wood-Derived Biochar for Supercapacitor Electrodes

**DOI:** 10.3390/bioengineering12080841

**Published:** 2025-08-04

**Authors:** Layal Abdallah, Chantal Gondran, Virginie Monnier, Christian Vollaire, Naoufel Haddour

**Affiliations:** 1 Univ Lyon, Ecole Centrale de Lyon, INSA Lyon, Université Claude Bernard Lyon 1, CNRS, Ampère, UMR5005, 69130 Ecully, France; layal.abdallah@ec-lyon.fr (L.A.); christian.vollaire@ec-lyon.fr (C.V.); 2 Département de Chimie Moléculaire UMR CNRS 5250, Université Grenoble Alpes, CNRS, 38000 Grenoble, France; chantal.gondran@univ-grenoble-alpes.fr; 3 Univ Lyon, Ecole Centrale de Lyon, INSA Lyon, Université Claude Bernard Lyon 1, CPE Lyon, CNRS, INL, UMR5270, 69130 Ecully, France; virginie.monnier@ec-lyon.fr

**Keywords:** cedar wood, biochar, pyrolysis temperature, binder, chitosan, specific capacitance, energy storage, electrode stability, supercapacitors

## Abstract

This study examines the effect of temperature during pyrolysis on the capacity of cedar wood-derived biochar to be employed as a sustainable electrode material for supercapacitors. Cedar wood-derived biochars were produced at different temperatures of 800 °C, 900 °C, 1000 °C and 1100 °C and fully characterized in terms of their structural, physicochemical and electrochemical properties, including specific surface area, hydrophobicity, electrical conductivity, and surface functional groups. The results indicated that the cedar wood biochar obtained through pyrolysis at 900 °C (BC900) provided optimal electrical conductivity, hydrophobicity, and porosity characteristics relative to the other cedar wood biochars produced by pyrolysis at 800 °C to 1100 °C. Specifically, when compared to commercial activated carbon (AC), BC900 provided half the specific capacitance at a current density of 1 A g^−1^ and indicated that there is more potential for improvement with further activation and doping. The influence of the binder (either polyvinylidene fluoride (PVDF) or chitosan) in combination with conductive carbon black (CB) was also examined. Electrodes fabricated with PVDF binder showed higher specific capacitance, while biochar electrodes made from CB and chitosan (BC900/CB/chitosan) showed better electrical conductivity, wettability, and good electrochemical stability with >95% capacity retention even after 10,000 cycles.

## 1. Introduction

The global demand for new, innovative, and efficient methods of energy storage is increasing due to global reliance on renewable energy sources with the goal of reducing greenhouse gas emissions [1]. Supercapacitors are enhanced by having a high power density, fast charge and discharge rates, and long-cycle life. These features enable them to act as a bridge, since they also benefit from the properties traditional capacitors and batteries in rapid energy storage as well as delivery [2]. One of the most important parts of a supercapacitor is the electrode, as it profoundly affects the energy storage mechanism. Traditional electrode materials are mostly carbon based, and include carbon nanotubes (CNTs), graphene, and activated carbon. These materials and their composites are selected due to their excellent conductivity and high surface area, as well as their electrochemical and chemical stability [3]. However, these materials are generally derived from fossil resources and often require energy-intensive and costly production processes [4]. One promising direction in supercapacitor research involves the use of biochar for supercapacitors, a carbon-rich material derived from biomass through a thermochemical conversion process known as pyrolysis [5]. In fact, biochar is preferred over traditional carbon-based materials due to its abundant availability and favorable material features. It is also a good candidate for supercapacitor electrodes because of its cost-effectiveness [6]. In addition, the environmental costs associated with biochar production and use are considerable, since, together with other mitigation activities, these processes alone could potentially sequester up to 0.3 to 2 billion tons of CO_2_ a year by 2050 [7]. Biochar is naturally self-sustainable, resulting from waste biomass such as agricultural byproducts, wood, and food waste; this makes it it economically reasonable as well as environmentally friendly [8,9]. The high porosity and large surface area of biochar are advantageous for applications related to energy storage [10,11,12]. Several parameters during pyrolysis affect the unique features of biochar, such as its porosity, conductivity, and surface chemistry [13,14]. Biochars produced from higher pyrolysis temperatures typically a possess greater surface area and less oxygenated functional groups, making them more hydrophobic and conductive [15,16]. The selection of biomass feedstock also plays a crucial role in determining the electrochemical activity of the resulting biochar. For example, biochars produced from lignocellulosic biomass such as wood are known to have high structural stability while possessing considerable a specific surface area, making them useful for supercapacitors [17,18]. However, biochar materials may suffer from batch-to-batch variability due to inconsistent feedstock composition, and often exhibit limited surface area without activation, requiring careful tuning for advanced electrochemical applications. Electrochemical performance, especially specific capacitance, is not only influenced by surface area but also by the pore structure and available active sites in the biochar skeleton [19,20]. While micropores (<2 nm) are effective for ion adsorption, mesopores (2–50 nm) are more suited for ion movement within the electrode material, which is vital for high-performance systems with fast charge–discharge cycles [21]. The development of hierarchical pore structures within biochar pores has therefore been the focus of recent studies, since it can achieve a more favorable combination of micro- and mesopores and increase both the capacitance and the rate capability. Such porosity is usually introduced by activation methods, for example chemical activation with KOH or physical activation with CO_2_, which have been shown to increase specific capacitance and cycle stability [22,23]. In an attempt to enhance the electrochemical characteristics of biochar, researchers have studied heteroatom doping for the substitution of the carbon matrix by elements like nitrogen, sulfur and phosphorus [24,25,26]. The resulting changes may lead to an increase in active site density, enhanced wettability, and improved electron conductivity, which all lead to higher capacitance values. As an example, nitrogen-doped biochars have been proven to show significantly increased electrochemical performance due to the existence of pyridinic and graphitic nitrogen species, which incur additional pseudocapacitance [27]. Co-doping with sulfur and nitrogen has also been proposed to have synergistic impacts on ion transport and electron mobility, as seen in peanut shell-derived biochar electrodes, which have been able to produce specific capacitances of 224 F g^−1^ [2]. Although there has been some progress in biochar-based supercapacitors, attention has not been paid to meticulously understanding the effects of various pyrolysis temperatures on the characteristics of biochar from different biomass origins. This study attempts to address this gap by focusing on cedar wood biochar produced at various temperatures, 800 °C, 900 °C, 1000 °C and 1100 °C, corresponding to BC800, BC900, BC1000 and BC1100, respectively. The electrochemical performance in supercapacitor applications is analyzed to find the optimal pyrolysis temperature by examining surface area, porosity, and functional groups. Furthermore, this research evaluates the role of various binders, especially chitosan, in improving electrode conductivity, wettability, and stability over time. This research provides fundamental knowledge toward the environmentally sustainable development of high-performance biochar-based supercapacitors and complements research oriented toward developing green materials.

## 2. Materials and Methods

### 2.1. Materials

Cedar wood was obtained from ManoMano, France. Super P conductive carbon black (CB) was purchased from Alfa Aesar, Strasbourg, France. The polypropylene (PP) membrane (Celgard^®^ 3501) was obtained from Celgard, Sélestat, France. All other chemicals used were purchased from Merck, France.

### 2.2. Biochar Preparation

Cedar wood was cut into pieces measuring 70 mm × 50 mm × 3 mm, washed with deionized water to remove impurities, and dried in an oven at 60 °C for at least 24 h. The prepared wood pieces were placed in combustion boats and pyrolyzed in a tubular furnace (model RSH 50/500/13, Nabertherm, Berlin, Germany) under an inert nitrogen atmosphere. The air inside the ceramic tube was first evacuated using a vacuum pump, and nitrogen gas was then injected at a flow rate of 100 L h^−1^ using a controlled gas supply system (controller B 410, Nabertherm, Berlin, Germany). High-purity nitrogen (99.999%) was used to ensure an inert atmosphere and prevent oxidative degradation during pyrolysis. The furnace was heated from room temperature to the target pyrolysis temperatures of 800 °C, 900 °C, 1000 °C, and 1100 °C, at a heating rate of 5 °C min^−1^. Once the target temperature was reached, it was maintained for 2 h to ensure complete pyrolysis. After the process, the furnace was cooled to ambient temperature to preserve the structural integrity of the resulting biochar. The biochar samples were then collected, labeled as BC800, BC900, BC1000, and BC1100 according to their respective pyrolysis temperatures, and stored in sealed glass containers to prevent contamination. The average biochar yield relative to the initial wood mass was ~26% for pyrolysis temperatures between 800 and 1000 °C.

### 2.3. Physicochemical Characterization of Biochar

The hydrophilic and hydrophobic characteristics of the biochar samples were evaluated using contact angle measurements. Contact angle measurements were carried out on flat-surfaced biochar monoliths rather than powders. Biochar pieces with naturally flat areas were selected for each condition, and gently sanded if needed to ensure surface planarity. This preparation minimized errors associated with the shadow method. An illustrative photograph of the droplet on the surface is provided in Supplementary Information (Figure S1). A 5 µL droplet of deionized water was carefully deposited on the biochar surface at room temperature, and the contact angle was measured using a contact angle measurement device (DMs-401, Kyowa Interface Science, Saitama, Japan). To compute the mean contact angle, the averaged angles were measured from both sides, and this procedure was performed for several regions of every sample for precision. The functional groups present in the biochar samples were analyzed through the Fourier transform infrared (FTIR) spectrometer (Nicolet 6700 FTIR spectrometer, Thermo Fisher Scientific, Waltham, MA, USA). For this purpose, 5 mg of biochar powder was finely ground and mixed with 120 mg of KBr powder. The mixture was dried at 200 °C to remove any residual moisture and then pressed into pellets. FTIR spectra were recorded in the range of 4000–500 cm^−1^ using an FTIR spectrometer in transmission mode (Nicolet 6700, Thermo Fisher Scientific, Waltham, MA, USA). The pH of the biochar samples was determined by dispersing 250 mg of biochar powder in 20 mL of deionized water, together with 10 glass beads in Falcon tubes. The dispersion was homogenized by sonication for 5 min and allowed to rest for 4 min. pH measurements were performed using a pH meter (OpH218, Origalys, Rillieux-la-Pape, France), with each sample measured in triplicate to ensure reproducibility. Raman spectroscopy was conducted to assess the crystallinity and structural order of the biochar samples. Spectra were collected using a Renishaw Invia Raman Microscope (Renishaw, Wotton-under-Edge, UK) with a 532 nm laser excitation wavelength. In order to detect possible heterogeneities in crystallinity, different parts of each sample were examined.

### 2.4. Structural and Morphological Characterization of Biochar

The specific surface area and porosity of the biochar samples were measured on 100 mg of crushed biochar using a specific surface area analyzer (NOVA4200E, Cantar Instruments, Odelzhausen, Germany). As confirmed by previous studies, CO_2_ and N_2_ adsorption measurements are complementary for characterizing porous carbon materials. CO_2_ adsorption at 273 K enables effective probing of ultramicropores (<1 nm) due to the higher diffusivity and stronger interaction of CO_2_ molecules with narrow pores, while N_2_ adsorption at 77 K is better suited for characterizing mesopores and larger pore structures. Combining both methods provides a more comprehensive understanding of pore architecture and accessibility in biochar materials [28,29]. N_2_ adsorption–desorption isotherms were measured at 77 K in the relative pressure interval of 0.05–0.3 (p/p_0_) to evaluate mesopore and macropore characteristics using the BET (Brunauer–Emmett–Teller) method for surface area and the BJH (Barrett–Joyner–Halenda) method for mesopore volume and pore size distribution. For samples exhibiting poor N_2_ accessibility (particularly those pyrolyzed at ≥900 °C), CO_2_ adsorption isotherms were recorded at 273 K to probe ultramicropores (<1 nm). The BET surface area was calculated from the CO_2_ isotherms using the pressure range 0.01–0.03 p/p_0_, and the micropore size distribution was derived using the Horvath–Kawazoe (HK) method. This method provides insights into the surface area and porosity of the biochar, which are critical for evaluating its suitability for energy storage applications. The resulting isotherms are provided in the Supplementary Information (Figure S2). As shown in Table 1, Supplementary Figure S3 and Supplementary Table S1, a drastic decrease in BET surface area was observed, from 370 m^2^ g_−1_ for BC800 to nearly negligible values for biochars produced at higher temperatures (BC900–BC1100). This pronounced decline is attributed to the limited diffusion of N_2_ molecules into ultramicropores at cryogenic temperatures (77 K), especially in samples where the surface becomes increasingly non-polar and chemically inert due to progressive carbonization. This phenomenon is particularly significant in high-temperature biochars with a high degree of graphitization and reduced surface functionalization [1,2]. To overcome this limitation and qualitatively assess microporosity in these high-temperature samples, CO_2_ adsorption was performed at 273 K. CO_2_, owing to its smaller kinetic diameter and stronger interaction with carbon surfaces, enables more reliable access to ultramicropores under these conditions. The morphology and structural features of the biochar were characterized by field emission scanning electron microscopy (FE-SEM) using a TESCAN MIRA-3 microscope (TESCAN-ORSAY, Brno, Czech Republic). This analysis was conducted to investigate the porosity, structural layout, and surface texture of the biochar, providing essential data on the material’s microstructure. Before SEM imaging, the biochar samples were carefully cleaned to remove any residual debris, dried at ambient conditions, and mounted on aluminum stubs with conductive carbon tape to ensure proper imaging.

### 2.5. Electrical Characterization of Biochar

The electrical resistivity of biochar was determined using two complementary methods. The first method employed the four-point probe in-line technique, which minimizes the influence of parasitic contact resistances. Preselected biochar samples with dimensions of 40 mm × 10 mm and a thickness ranging from 0.5 mm to 1.5 mm were sanded to ensure flat surfaces. Measurements were conducted using aligned tungsten carbide probes with a radius of 125 μm and a pitch of 1.27 mm, connected to an S-302 stand (Signatone, Grenoble, France). The resistivity *ρ* (Ω m) for an infinite sheet of finite thickness was calculated using the following equation:

(1) $$\rho =R_{sh-2D}^{line}\times t\;\times F_{1}\left(\frac{t}{s}\right)=\left[\frac{\pi }{\ln\left(2\right)}\times R\right]\times t{\times F}_{1}\left(\frac{t}{s}\right)$$

where $R_{sh-2D}^{line}=\frac{\pi }{ln\left(2\right)}R$ represents the sheet resistance of an infinite two-dimensional sheet, $R=\frac{V}{I}$ is the resistance measured between two probes, *t* is the thickness of the sample, and *F*_1_ is a correction factor that accounts for the finite thickness of the sample, depending on the ratio $\frac{t}{s}$ with *s* the distance between two probes. *F*_1_ is a dimensionless factor; its correction is negligible when *t* ≪ *s* (*F*_1_→1) and decreases while *t* increases. *F*_1_ is expressed according to the following formula [30]:

(2) F1ts=ln2lnsinhtssinht2s

The second method involved direct measurement of the resistivity of biochar powders and biochar-based electrodes using the internal resistance feature of an Origalys potentiostat. In this approach, the ohmic resistance *R* (Ω) of the sample was measured, and the resistivity *ρ* (Ω m) was calculated using the following formula: $\rho =R\;\times \;\frac{S}{l}$, where *S* (m^2^) is the cross-sectional surface area of the electrodes, and *l* (m) is their thickness.

### 2.6. Preparation of Biochar-Based Electrodes and Assembly of Supercapacitor

Electrodes were prepared by grinding a mixture of biochar, carbon black (CB), and a binder in a weight ratio of 70:20:10. The binders used were polyvinylidene fluoride (PVDF) and chitosan with PVDF in ethanol 50%. For the PVDF electrodes, ethanol was used to homogenize the mixture before being drop-casted onto aluminum current collectors. Room-temperature drying was then utilized to help with adhesion. The electrodes, using chitosan as a binder, utilized a 2% (*w*/*v*) chitosan solution that was prepared by dissolving chitosan powder in a 2% (*v*/*v*) aqueous acetic acid solution [31]. This solution was then magnetically stirred at room temperature for 12 h until a uniform pale-yellow mixture was created that would then be used for the binding phase of the biochar–CB mix. The resulting paste was homogenized and drop-casted onto aluminum current collectors, and then left to dry under ambient conditions. The resulting 0.01 g electrode pastes were coated onto an aluminum collector (0.11 cm^2^). Therefore, the loading of the active electrode was 0.06 g cm^−2^. The electrodes were assembled into a symmetric two-electrode supercapacitor cell structure in the Swagelok configuration. Each cell was built with two electrodes, based on the biochar, that were interspaced with a polypropylene (PP) separator (Celgard^®^ 3501, Celgard, Sélestat, France) so that the electrodes could not short-circuit. For thorough electrolyte saturation, the PP separator was immersed in 1 M Na_2_SO_4_ aqueous electrolyte for an hour before assembly. Each cell was sealed post-assembly and prepared for the electrochemical tests.

### 2.7. Electrochemical Characterization of Biochar

The electrochemical properties of the biochar-based electrodes were evaluated using an OrigaFlex potentiostat workstation (Origalys, Rillieux-la-Pape, France). Cyclic voltammetry (CV) measurements were performed in a potential window of −0.8 V to 0.8 V using 1 M Na_2_SO_4_ as the electrolyte. Various scan rates (10, 20, 100, 250, and 500 mV s^−1^) were employed to analyze the capacitive behavior and rate capability of the electrodes. Galvanostatic charge–discharge (GCD) measurements were conducted at current densities varying between 1 and 5 A g^−1^ to assess the specific capacitance and energy efficiency of the electrodes. All electrochemical experiments were carried out at ambient temperature, and each measurement was repeated to ensure reliability and reproducibility. According to the data of GCD curves, the specific capacitance of the electrodes are calculated using Equation (3):

(3) $$C_{sp}=\frac{I\;\times \;\Delta t}{m\;\times \;\Delta V}$$

where *C* (F g^−1^) is the specific capacitance of the material, *I* (A) is the discharge current, Δ*t* (s) is the discharge time, *m* (g) is the mass of the active material in the electrode, and Δ*V* (V) is the potential window of the electrode.

## 3. Results and Discussion

### 3.1. Effect of Pyrolysis Temperature on the Properties of Cedar Wood-Based Biochar

The examination of cedar wood biochar produced at various temperatures of 800, 900, 1000 and 1100 °C showed trends in resistivity, specific surface area, contact angle, and pH that determined the biochar’s effectiveness in supercapacitor use, as shown in Table 1. The resistivity of the biochar decreased significantly, from 18 × 10^−3^ Ω m at 800 °C to just 3.6 × 10^−3^ Ω m at 1100 °C, which suggests an improvement in electrical conductivity. This drop in resistivity is consistent with the findings of other studies, where increased carbonization and the removal of oxygenated groups resulted in a more conductive graphitic structure at higher temperatures [2,16]. An increase in conductivity is vital for the supercapacitor’s electrodes, resulting in rapid charge transfer within the material.

The specific surface area changed with temperature, as the specific surface area was at its maximum, 454 m^2^ g^−1^, at 800 °C, and then dropped to 385 m^2^ g^−1^ at 900 °C; it then increased to 431 m^2^ g^−1^ at 1000 °C and finally leveled out at 425 m^2^ g^−1^ at 1100 °C. The decrease in surface area observed at 900 °C may have been a sign of thermal destruction of micropores, while the subsequent increase in surface area observed at higher temperatures may have resulted from the rearrangement of the structure through mesopore pore development. Other investigations have also found such transitions in pore structure with elevated pyrolysis temperatures, and comparable definitions have been proposed based on biochar derived from different biomass sources [32,33].

Contact angle measurements demonstrated an increase in this parameter from 26° at 800 °C to approximately 53° at 1000 °C and 1100 °C, indicating a transition toward a more hydrophobic surface with rising temperatures. This increase in hydrophobicity corresponds to a loss of hydrophilic functional groups, such as hydroxyl and carboxyl groups, consistent with previous studies that observed similar changes in biochar produced at high temperatures [34].

**Figure 1:**
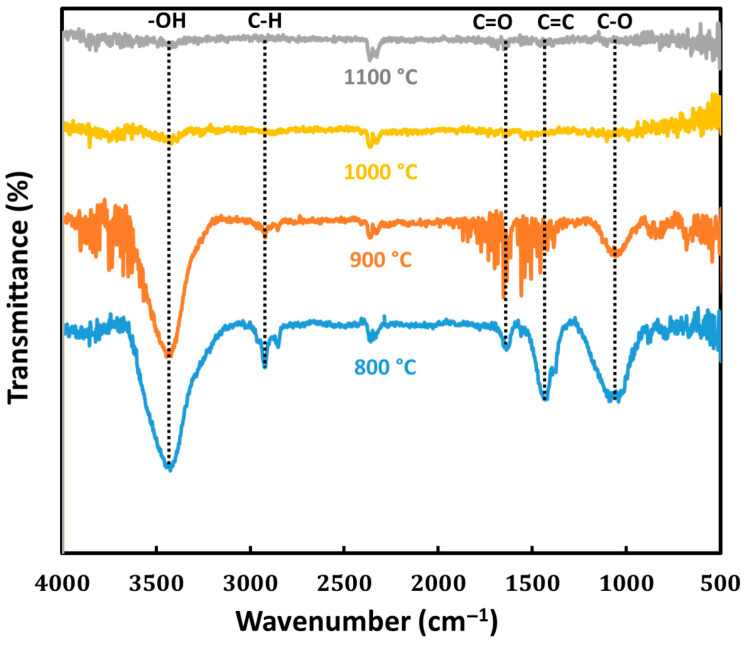
FTIR spectra of cedar wood-based biochars obtained at pyrolysis temperatures of 800 °C (blue), 900 °C (orange), 1000 °C (yellow), and 1100 °C (gray).

This is in agreement with the results obtained from FTIR spectroscopy (Figure 1). The infrared spectra show that at 800 °C (BC800), certain characteristic functional groups are visible, which include a broad band near 3400 cm^−1^ relative to the O–H bond stretching (hydrogen bonding) of the hydroxyl of a carboxylic acid or alcohol, a sharp peak relative to C–H aliphatic at around 2850 cm^−1^, C=C stretching at 1450 cm^−1^, C=O (carbonyl) stretching at 1700 cm^−1^, and a peak around 1000 cm^−1^ corresponding to C–O stretching vibrations. These functional groups reflect the lignocellulosic structure of the biomass, which is composed of cellulose, hemicellulose, and lignin. At 900 °C (BC900), it was possible to notice peaks at the same wave numbers but with a lower intensity, indicating a reduction in these functional groups. If the pyrolysis temperature is increased further, these functional groups would be lost due to the breaking of bonds caused by dehydroxylation, decarboxylation, and decarbonylation reactions, which would result in structures richer in carbon. This reduction in hydrophilic functional groups, as seen in the FTIR spectra, explains the increased hydrophobicity in the measurements of the contact angles. A moderate level of hydrophobicity can minimize moisture, but when the hydrophobicity becomes extremely high, interactions with water-based electrolytes may be hindered, which is not ideal for electrochemical activity.

Even though increased pyrolysis temperatures resulted in a reduction in hydrophilic functional groups which increases the material’s hydrophobicity, the biochar’s pH level was consistently maintained around 7.8–7.9 across all temperatures. This indicates a neutral character. This particular stability suggests that while surface functional groups decrease, the greater proportions of acidic and basic functionalities contained within the biochar structure are neutralized and remain unchanged [35]. Furthermore, the FTIR results corroborate this, showing the disappearance of specific functional groups without significantly altering the biochar’s inherent pH. A neutral pH is advantageous for maintaining chemical stability in electrochemical devices because it does not initiate undesired reactions with the electrolyte which may lead to poor performance. This illustrates how critically important the choice of feedstock is in producing biochar with specific desirable chemical properties, as the feedstock material’s composition predominantly determines the characteristics of the final product.

**Figure 2:**
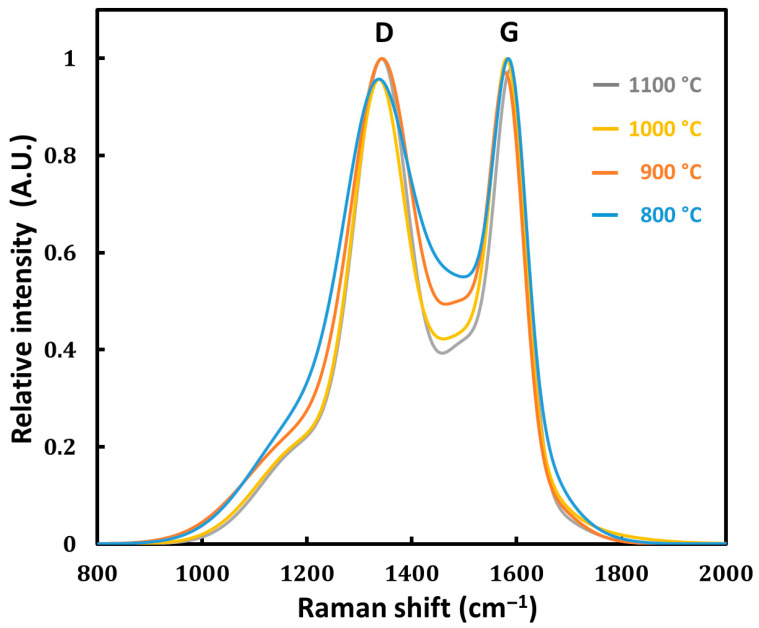
Raman spectra of cedar wood-based biochar prepared at pyrolysis temperatures of 800 °C (blue), 900 °C (orange), 1000 °C (yellow), and 1100 °C (gray). The **G band** represents the vibration of ordered sp^2^ carbon atoms (graphitic structure), while the **D band** indicates defects or disorder in the carbon lattice.

Raman spectroscopy analysis of cedar wood biochar pyrolyzed at temperatures of 800, 900, 1000, and 1100 °C provides insights into the evolution of the graphitic structure and defect density within the carbon matrix (Figure 2). The spectra display two primary peaks: the D band around 1350 cm^−1^, associated with structural defects and disordered carbon, and the G band near 1580 cm^−1^, indicative of sp^2^-bonded carbon atoms in graphitic domains. As pyrolysis temperature increases from 800 °C to 1100 °C, the valley between the D and G bands becomes deeper and more pronounced. This feature is indicative of a gradual transition toward a more ordered carbon structure, associated with the formation of extended sp^2^-hybridized domains and a reduction in defect density. The visual evolution of the valley region aligns with previous studies showing that higher pyrolysis temperatures promote carbon atom reorganization and enhanced graphitic ordering [36]. This trend complements the FTIR findings, which showed a loss of specific functional groups due to thermal decomposition, and aligns with the observed increase in hydrophobicity from contact angle measurements. This supports research findings that biochars subjected to high pyrolysis temperatures exhibit greater crystallinity and reduced defect density—attributes advantageous for conductive applications. Overall, Raman analysis confirms that pyrolysis at 1000 °C and above enhances the graphitic nature of cedar wood biochar, while pyrolysis at 900 °C results in a balance between defect presence and structural ordering. This comprehensive analysis underscores the interconnected nature of chemical composition, surface characteristics, and structural evolution in determining a biochar’s suitability for supercapacitor applications.

The resistivity of the biochar decreased significantly with increasing pyrolysis temperature, indicating an improvement in electrical conductivity (Table 1). Specifically, the resistivity dropped from 18 × 10^−3^ Ω m at 800 °C to 5 × 10^−3^ Ω.m at 900 °C, further decreased to 3.7 × 10^−3^ Ω m at 1000 °C, and stabilized at 3.6 × 10^−3^ Ω m at 1100 °C. This reduction in resistivity is closely linked to the structural and compositional changes observed through FTIR spectroscopy, contact angle measurements, and Raman spectroscopy. FTIR spectra reveal that higher pyrolysis temperatures lead to the disappearance of oxygen-containing functional groups such as hydroxyl and carboxyl groups, due to dehydroxylation and decarboxylation reactions. This loss of polar functional groups reduces the number of electron-scattering sites and contributes to increased electrical conductivity. The increase in hydrophobicity observed in the contact angle measurements, from 26° at 800 °C to approximately 53° at 1000 °C and 1100 °C, corroborates the FTIR findings, indicating a decrease in surface polarity and suggesting a higher degree of carbonization. Raman spectroscopy provides additional insights into the structural evolution of the biochar, indicating a reduction in defect density and an enhancement in graphitic ordering. The decreased peak separation between the D and G bands suggests the formation of larger sp^2^-hybridized carbon domains, which facilitate electron transport within the carbon matrix. Collectively, these analyses demonstrate that higher pyrolysis temperatures promote increased carbonization, the removal of oxygenated functional groups, and the development of a more ordered graphitic structure. These changes result in a significant decrease in resistivity and the improved electrical conductivity of the biochar. Enhanced conductivity is crucial for supercapacitor electrodes, allowing for efficient charge transfer within the material, thereby improving electrochemical performance.

**Figure 3:**
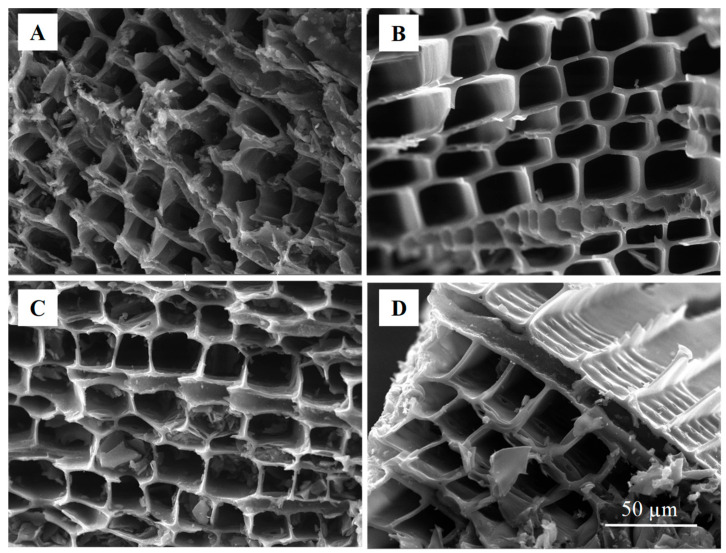
SEM images of cedar wood-based biochars prepared at various pyrolysis temperatures: (**A**) 800 °C; (**B**) 900 °C; (**C**) 1000 °C; (**D**) 1100 °C. Scale bar: 50 µm.

Scanning electron microscopy (SEM) images of the BC800, BC900, BC1000, and BC1100 samples display macroporous structures with a honeycomb-like appearance (Figure 3). These organized macropores are bordered by thin carbon walls separated by distances ranging from 10 to 40 μm. These walls correspond to the carbonized cell walls of the woody biomass, which are predominantly composed of cellulose, hemicellulose, lignin, and pectin [37]. Lignin, being the most resistant component to thermal decomposition, helps preserve the cell wall structure up to certain pyrolysis temperatures, as previously noted [38].

The morphological analysis we conducted via SEM revealed slight differences in the pore structure of cedar wood-based biochar depending on the pyrolysis temperature. At the lower pyrolysis temperature of 800 °C, the biochar structure exhibited irregular and relatively closed macropores (Figure 3A), indicating partial decomposition of the lignocellulosic structure, consistent with observations in similar studies on low-temperature biochar [39]. As the temperature increased to around 900 °C, the structure became more uniform (Figure 3B), with well-developed macropores that provided optimal pathways for ion transport. The difference between the results of this study and those of our previous study using the same pyrolysis temperature (900 °C) can be attributed to the age and condition of the cedar wood. In our previous study, aged cedar biomass was used, which led to a collapse of the honeycomb-like structure at high temperatures, significantly reducing the surface area. In the present work, fresh cedar wood was used, preserving the structural integrity during pyrolysis and allowing for higher surface development. This enhanced porosity improved the interaction between electrolyte ions and the electrode surface, a crucial factor for supercapacitor applications, as noted in studies showing increased electrochemical performance with enhanced pore connectivity [40]. Although full porosity distribution analysis was not performed in this study, the CO_2_ adsorption isotherms are included in Supplementary Information (Figure S2) and show characteristics consistent with microporous materials. At higher temperatures, close to 1000 °C, the biochar develops well-oriented channels (Figure 3C,D), but part of its structure collapses due to the excessive thermal treatment. This trend is similar to findings obtained from other biomass-derived biochars, where high pyrolysis temperatures lead to partial structural collapse. This progression in morphology, from poorly connected pores to optimal porous structures and eventually to partial degradation at higher temperatures, highlights the necessity of optimizing the pyrolysis temperature. Achieving a balance between structural stability and high surface area is essential for producing a biochar suitable for supercapacitor applications.

### 3.2. Electrochemical Characterization of Biochar-Based Electrodes

Electrodes composed of biochar combined with carbon black (CB) and different binders were further characterized through contact angle measurements and electrical resistivity tests to evaluate the effect of conductive additives and binders on their properties (Table 2). The resistivity measurements indicated a decrease when biochar was combined with carbon black and polyvinylidene fluoride (PVDF) binder compared to when biochar was used alone. Specifically, the resistivity of the BC800-based electrode decreased from 8 × 10^−3^ Ω m for biochar alone to 6.43 × 10^−3^ Ω m when combined with CB and PVDF. Similarly, BC900/CB/PVDF and BC1000/CB/PVDF electrodes exhibited resistivities of 3.35 × 10^−3^ Ω m and 2.75 × 10^−3^ Ω m, respectively. This improvement in electrical conductivity can be attributed to the percolation network formed by carbon black, which enhances electron transport within the electrode matrix [41]. However, contact angle measurements revealed varied hydrophobicity behavior among the electrodes. The BC800/CB/PVDF electrode exhibited a contact angle of 127°, significantly higher than the 26° angle measured for BC800 alone, indicating increased hydrophobicity due to the presence of PVDF. Similarly, the BC900/CB/PVDF electrode showed a contact angle of 116°. Increased hydrophobicity can hinder ion transport in aqueous electrolytes, potentially affecting electrochemical performance. Interestingly, the BC1000/CB/PVDF electrode displayed a drastic decrease in contact angle to 17 ± 6°, indicating a significant increase in hydrophilicity, which is difficult to explain based on the current understanding. This unexpected decrease in hydrophobicity for the BC1000/CB/PVDF may be attributed to several plausible factors. One hypothesis is that at higher pyrolysis temperatures, structural changes in the biochar expose more hydrophilic sites. Although FTIR spectroscopy indicated a reduction in oxygen-containing functional groups with increasing temperature, new defects or edge sites created at higher temperatures may possess hydrophilic characteristics. Another possibility is that the increased graphitic ordering at 1000 °C, as evidenced by Raman spectroscopy, alters the surface energy characteristics, resulting in enhanced wettability. Additionally, interactions between the biochar, carbon black, and PVDF binder might differ at higher pyrolysis temperatures, potentially affecting the distribution and coating efficiency of PVDF on the biochar surface, leading to more exposed hydrophilic biochar sites. Further investigation is necessary to fully understand the mechanisms behind this observation. Surface chemical analysis techniques, such as X-ray photoelectron spectroscopy (XPS), could provide insights into changes in surface functional groups or elemental composition that may explain the increased hydrophilicity. The enhanced wettability of the BC1000/CB/PVDF electrode could be beneficial for ion transport in aqueous electrolytes, potentially improving electrochemical performance. The increased hydrophobicity could hinder ion transport in aqueous electrolytes, potentially affecting electrochemical performance. To mitigate the issue of hydrophobicity in electrodes containing PVDF, chitosan, a binder with more hydrophilic properties, was used as a substitute for PVDF. The BC900/CB/chitosan electrode demonstrated a significant decrease in resistivity to 0.61 × 10^−3^ Ω m, compared to 3.35 × 10^−3^ Ω m for the BC900/CB/PVDF electrode, indicating enhanced electrical conductivity. Additionally, the contact angle decreased from 116 ± 8° to 63 ± 4°, reflecting increased hydrophilicity. This suggests that chitosan not only enhances the wettability of the electrodes by improving their hydrophilic character but also boosts their electrical conductivity. According to the study reported by Salleh et al., using chitosan as a biopolymer binder for graphene-based supercapacitor electrodes significantly improves the distribution of conductive materials and promotes electrode homogeneity [42]. Chitosan forms a uniform and continuous network within the electrode, reducing contact resistance and facilitating efficient electron transport pathways. Additionally, its inherent ionic conductivity further augments the overall conductivity of the electrode, promoting better electrochemical performance. Furthermore, the study indicates that chitosan’s ability to form strong interactions with carbon-based materials enhances the dispersion of conductive particles and establishes more efficient conductive networks within the electrode. The protonated amino groups of chitosan can form electrostatic interactions with the negatively charged surfaces of carbon materials, such as graphene or biochar, improving particle adhesion and connectivity. This interaction minimizes void spaces and ensures tighter packing of the conductive particles, which leads to reduced resistance and enhanced charge transport efficiency. The resulting improvement in electrical properties is critical for achieving high specific capacitance and superior energy storage performance in supercapacitors. In addition, chitosan’s hydrophilic nature ensures better compatibility with aqueous electrolytes, further supporting its role as an effective and sustainable alternative to traditional synthetic binders. Furthermore, the resistivity and contact angle values of the BC900/CB/chitosan electrode are comparable to those for an electrode with commercial activated carbon (AC/CB/PVDF and AC/CB/chitosan); both types of electrode resistivities of 0.61 × 10^−3^ Ω m and 0.62 × 10^−3^ Ω m and contact angles of 44 ± 6° and 39 ± 2°, respectively. This parity suggests that biochar pyrolyzed at 900 °C, when combined with carbon black and chitosan binder, exhibits properties similar to those of commercial activated carbon-based electrodes. Therefore, integrating carbon black and selecting an appropriate binder can significantly influence the electrical and wetting properties of biochar-based electrodes. Using chitosan as a binder helps increase both electrical conductivity and hydrophilicity, likely due to its capacity to form a more hydrophilic and conductive network within the electrode. These improvements are crucial for supercapacitor applications, where efficient electron transport and ion diffusion are essential for high performance.

### 3.3. Electrochemical Characterization of Biochar-Based Supercapacitors

**Figure 4:**
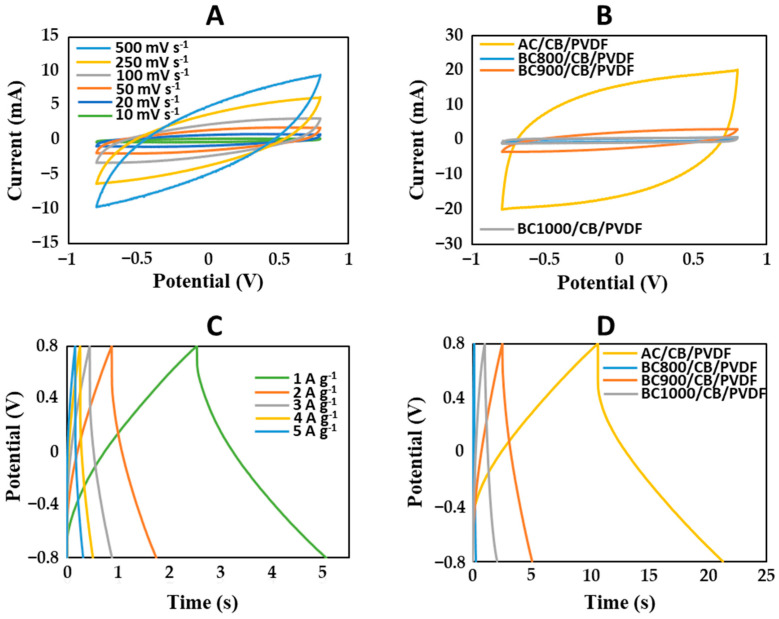
(**A**) CV curves of BC900/CB/PVDF electrodes at various scan rates from 10 to 500 mV·s^−1^; (**B**) CV curves of cedar wood biochar-based electrodes combined with CB and PVDF at 100 mV·s^−1^; (**C**) GCD curves of BC900/CB/PVDF-based electrodes at different current densities; (**D**) GCD curves of BC800/CB/PVDF, BC900/CB/PVDF, BC1000/CB/PVDF and AC/CB/PVDF electrodes at 1 A g^−1^.

Electrodes composed of biochar combined with carbon black (CB) and different binders were characterized further. The electrochemical performance of cedar wood-derived biochar-based supercapacitors was evaluated using cyclic voltammetry (CV) and galvanostatic charge–discharge (GCD) measurements in a two-electrode symmetric configuration with 1 M Na_2_SO_4_ as the electrolyte at ambient temperature (Figure 4). The performance of biochar-based supercapacitors was compared with supercapacitors using activated carbon (AC) as a conventional electrode material. The CV curves of BC900/CB/PVDF supercapacitors at scan rates ranging from 10 to 500 mV s^−1^ (Figure 4A) exhibit a quasi-rectangular shape, indicative of an electrical double-layer capacitance (EDLC) mechanism, without distinct redox peaks. This suggests that charge storage occurs predominantly through electrostatic ion adsorption at the electrode–electrolyte interface with non-faradaic reactions. When comparing the CV curves of BC800/CB/PVDF, BC900/CB/PVDF, BC1000/CB/PVDF, and AC/CB/PVDF supercapacitors at 100 mV s^−1^ (Figure 4B), the enclosed area of the AC-based electrode is the largest, followed by that of BC900/CB/PVDF, then that of BC1000/CB/PVDF and that of BC800/CB/PVDF. This observation implies that BC900 achieves a higher specific capacitance than BC800 and BC1000, possibly owing to an optimized microstructure that enhances porosity, electrical conductivity, and ion accessibility. The GCD curves further confirm the capacitive behavior of biochar-based electrodes, displaying nearly symmetric triangular profiles, consistent with the CV results (Figure 4D).

**Figure 5:**
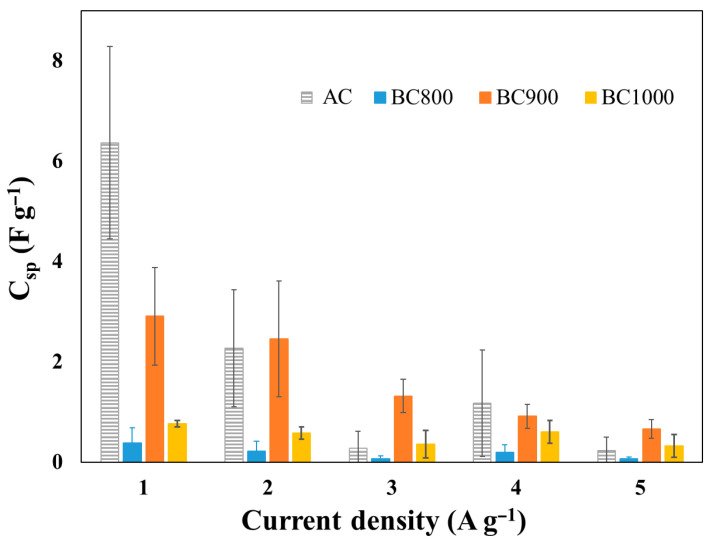
Specific capacities of BC800/CB/PVDF, BC900/CB/PVDF, BC1000/CB/PVDF and AC/CB/PVDF electrodes in a symmetric SC configuration.

The specific capacitance of BC800 electrodes was the lowest at all tested current densities (Figure 5). At 1 A g^−1^, BC800 electrodes demonstrated a specific capacitance of 2.6 F g^−1^, which is lower than that of BC900 (2.9 F g^−1^), while that of BC1000 electrodes reached 0.7 F g^−1^ (Figure 5). In comparison, commercial AC electrodes demonstrated a specific capacitance of 6.3 F g^−1^ at the same current density, which is comparable to previous results reported by Durajski et al. for supercapacitors fabricated from unmodified AC (1.33 F g^−1^) [43]. The inferior performance of BC800 can be attributed to its higher resistivity (18 × 10^−3^ Ω m), which is nearly three times greater than that of BC900 (5.3 × 10^−3^ Ω m), leading to increased internal resistance and reduced charge storage efficiency. A decline in specific capacitance was observed as the current density increased, likely due to ion diffusion limitations within the electrode’s micropores and mesopores. For instance, BC900 electrodes showed a specific capacitance of 2.9 F g^−1^ at 1 A g^−1^; this decreased to 0.6 F g^−1^ at 5 A g^−1^ (Figure 5), highlighting how charge transport dynamics affect electrode performance. However, a direct comparison with the reference electrode based on activated carbon (AC/CB/PVDF) shows that it achieves the highest specific capacitance (6.3 F g^−1^ at 1 A g^−1^), more than twice the capacitance obtained with BC900/CB/PVDF (2.9 F g^−1^). These specific capacitance values, although modest compared to those of activated carbon, are consistent with those from other studies involving non-activated biochars derived from lignocellulosic biomass [44,45,46]. This difference highlights the potential for further optimizing the porous structure and conductivity of biochar in order to compete with the performance of commercial activated carbon. Nonetheless, it is important to note that biochar-based electrodes offer significant advantages in terms of cost and sustainability, mainly due to the renewable origin of the biomass. Therefore, optimizing the pyrolysis conditions, carrying out chemical or physical activation, and performing heteroatom doping (e.g., nitrogen or sulfur) could further align the electrochemical properties of biochar with those of activated carbon, all while maintaining a more favorable environmental profile.

**Figure 6:**
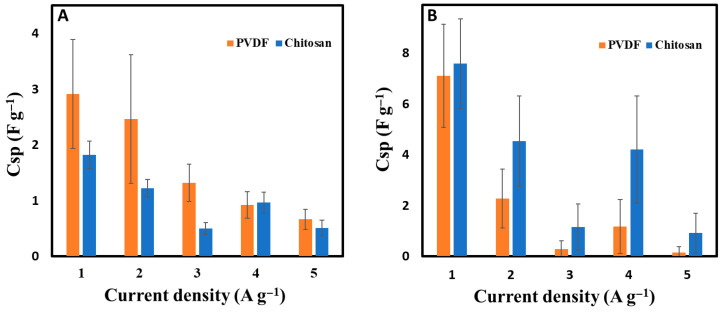
Specific capacitances of (**A**) BC900-based electrodes (PVDF vs. chitosan) and (**B**) AC-based electrodes (PVDF vs. chitosan) in a symmetric supercapacitor configuration.

The choice of binder significantly affected the electrical and electrochemical properties of the BC900 electrodes. Electrical conductivity increased sixfold when chitosan was used instead of PVDF (Table 2), and the hydrophobicity was reduced, as evidenced by a lower contact angle (63 ± 4° for chitosan vs. 116 ± 8° for PVDF, as shown in Table 2). This improved wettability suggests better electrolyte penetration and enhanced ion mobility within the electrode structure. However, despite its beneficial impact on conductivity and hydrophilicity, the specific capacitance of BC900 electrodes remained higher when PVDF was used as a binder. At 1 A g^−1^, PVDF-bound electrodes exhibited a capacitance 1.5 times higher than that of chitosan-bound electrodes, a difference that decreased to 1.3 times at 5 A g^−1^ (Figure 6A). Potential reasons for this discrepancy may include steric hindrance or particular interactions between the chitosan binder and biochar particles, possibly restricting pore accessibility and limiting overall charge storage. This effect has been observed in other studies, where the use of biopolymer-based binders resulted in the partial obstruction of porous structures in carbon electrodes, negatively affecting ion diffusion [42]. Interestingly, the opposite trend was observed for activated carbon (CA)-based electrodes, where chitosan outperformed PVDF as a binder across all current densities (Figure 6B). At 1 A g^−1^, the specific capacitance of chitosan-bound CA electrodes was slightly higher than that of PVDF-bound electrodes, and this difference became more pronounced as the current density increased. At 2 A g^−1^, the capacitance of chitosan-based electrodes was nearly twice that of PVDF-based ones, and at higher current densities, the chitosan binder enabled significantly better charge storage capability. This contrasting behavior between biochar-based and activated carbon-based electrodes suggests that the interaction between the binder and the electrode material plays a crucial role in electrochemical performance. While biochar is a less structurally ordered carbon material with a more heterogeneous porous network, the presence of chitosan may block some of the smaller pores or alter the ion transport pathways, leading to a slight reduction in capacitance compared to PVDF. On the other hand, activated carbon typically possesses a highly developed microporous and mesoporous structure, which may be better complemented by the presence of chitosan. Previous studies have shown that chitosan can increase specific capacitance by reducing the blocking on the surface of the electrode’s active materials [47,48]. Additionally, the strong interaction between the protonated amino groups of chitosan and the negatively charged surface of activated carbon could contribute to a more homogeneous electrode structure with improved electron transfer efficiency. This effect is less pronounced in biochar due to its more complex surface chemistry and lower graphitic ordering. Moreover, the higher capacitance retention of chitosan-bound activated carbon at elevated current densities suggests that chitosan enhances charge transfer kinetics and electrolyte wettability, reducing resistance and enabling more effective charge storage under high-power conditions. These findings highlight the importance of optimizing binder–material interactions to achieve the best electrochemical performance. While PVDF remains a superior binder for biochar-based electrodes in terms of capacitance, chitosan offers notable advantages when paired with activated carbon, providing an environmentally friendly and effective alternative for high-performance supercapacitors.

### 3.4. Electrochemical Stability of Biochar-Based Supercapacitors

Capacity retention is an essential metric for evaluating the long-term performance of supercapacitors, as it quantifies the energy storage device’s ability to maintain its initial capacity over multiple charge–discharge cycles. The cycling stability of cedar wood-derived biochar electrodes was assessed via GCD measurements over 10,000 cycles. The results show that supercapacitors fabricated with BC800/CB/PVDF, BC900/CB/PVDF, and BC1000/CB/PVDF electrodes retained approximately 93% of their initial capacitance, indicating excellent cycling stability and high reversibility during charge–discharge processes. This relatively high retention suggests that biochar-based electrodes preserve their structural integrity and conductivity under prolonged use, a key requirement for practical energy storage applications. Chitosan-based electrodes exhibited an even higher capacity retention of 95%, emphasizing the potential of chitosan as a sustainable and durable alternative to conventional polymer binders. This enhanced stability may be attributed to chitosan’s ability to form strong hydrogen bonds with the electrode material, improving mechanical cohesion and minimizing electrode degradation during repeated cycling. These findings confirm that biochar-based electrodes, particularly those incorporating chitosan as a binder, can achieve electrochemical stability on par with that of commercial activated carbon electrodes while offering lower production costs, environmental sustainability, and improved compatibility with aqueous electrolytes. Among the tested systems, the BC900/CB/chitosan electrode strikes an optimal balance between conductivity, capacitance, and long-term cycling performance, making it a compelling candidate for large-scale supercapacitor applications. Combining biochar with chitosan not only boosts electrochemical stability but also contributes to greener energy storage technologies by reducing dependence on fossil-based materials.

## 4. Conclusions

In this work, cedar wood-derived biochar produced at various pyrolysis temperatures (800–1100 °C) was investigated as a sustainable electrode material for supercapacitor applications. The results showed that the pyrolysis temperature exerts a strong influence on the physicochemical and electrochemical properties of biochar, including its specific surface area, electrical conductivity, and surface functional groups. Among the tested temperatures, biochar prepared at 900 °C (BC900) achieved a favorable balance between high electrical conductivity, moderate hydrophobicity, and adequate porosity, thereby delivering superior electrochemical performance compared to biochars prepared at 800 °C or 1000 °C. Nonetheless, direct comparison with activated carbon (AC) demonstrated that AC still outperforms BC900 in terms of specific capacitance, indicating room for the further optimization of biochar through activation techniques and potential heteroatom doping. Despite this performance gap, cedar wood biochar provides significant benefits in cost, sustainability, and renewability, warranting ongoing efforts to enhance its electrochemical properties. In parallel, the choice of binder (PVDF vs. chitosan) also impacted electrode properties. While PVDF-based electrodes generally exhibited higher specific capacitances, chitosan-based electrodes benefited from improved hydrophilicity, better ionic conductivity, and enhanced mechanical stability. Consequently, BC900 electrodes using chitosan showed particularly good electrical conductivity, favorable wettability, and capacity retention of up to 95% over 10,000 charge–discharge cycles, surpassing the long-term stability of some PVDF-based counterparts. Overall, these findings highlight the potential of cedar wood biochar as a cost-effective and environmentally friendly alternative to conventional activated carbons for supercapacitor electrodes. The combined effects of pyrolysis temperature optimization, conductive additives (carbon black), and a suitable binder (especially chitosan) lead to the obtention of electrodes with high stability, reasonable specific capacitance, and excellent cycling longevity. Future work could focus on the heteroatom doping (e.g., nitrogen, sulfur) of cedar wood biochar to further improve its electrochemical properties, as well as on exploring other biomass sources to develop a broader range of sustainable, high-performance electrode materials for energy storage applications.

## Figures and Tables

**Table 1:** Specific surface area, resistivity and contact angle measurements under different conditions.

Material	Specific Surface Area (N_2_, m^2^ g^−1^)	Specific Surface Area (CO_2_, m^2^ g^−1^)	Resistivity 10^−3^ (Ω m)	Contact Angle (°)	pH
BC800	370	454 ± 8	18 ± 1	26 ± 3	7.8 ± 0.1
BC900	1.7	385 ± 30	5.3 ± 0.1	51 ± 5	7.7 ± 0.1
BC1000	6.7	431 ± 40	3.70 ± 0.05	53 ± 4	7.9 ± 0.1
BC1100	7.3	425 ± 20	3.60 ± 0.06	53 ± 15	7.9 ± 0.2

**Table 2:** Resistivity and contact angle measurements with different conditions.

Electrode	Resistivity 10^−3^ (Ω m)	Contact Angle (°)
BC800/CB/PVDF	6.43 ± 0.05	127 ± 3
BC900/CB/PVDF	3.35 ± 0.01	116 ± 8
BC1000/CB/PVDF	2.75 ± 0.03	17 ± 6
AC/CB/PVDF	0.62 ± 0.10	44 ± 6
BC900/CB/chitosan	0.61 ± 0.21	63 ± 4
AC/CB/chitosan	0.68 ± 0.21	39 ± 2

## Data Availability

Data is contained within the article.

## Supplementary Materials

The following are available online at https://www.mdpi.com/article/10.3390/bioengineering12080841/s1, Figure S1: Procedure for contact angle measurement; Figure S2: CO_2_ adsorption isotherms for cedar wood-derived biochar samples pyrolyzed at 800, 900, 1000, and 1100 °C. Figure S3: N2 adsorption–desorption isotherms at 77 K for biochar samples obtained at different pyrolysis temperatures: (A) BC800, (B) BC900, (C) BC1000, and (D) BC1100. Table S1: Textural properties of biochar samples obtained at different pyrolysis temperatures, determined from nitrogen adsorption–desorption isotherms at 77 K. and from CO_2_ adsorption–desorption isotherms at 273 K.
