# Supplementary material for: Influence of Pyrolysis Temperature on the Properties and Electrochemical Performance of Cedar Wood-Derived Biochar for Supercapacitor Electrodes
Source: Bioengineering (Basel). 2025 Aug 4;12(8):841. doi: 10.3390/bioengineering12080841 (PMC12383750; doi:10.3390/bioengineering12080841)
Supplement: Supplementary file 1 [file bioengineering-12-00841-s001.zip › bioengineering-3649322-supplementary.pdf]

Supplementary Materials

S1. Procedure for contact angle measurement

Procedure for contact angle measurement of cedar wood-derived biochar. Raw cedar wood was cut and pyrolyzed under an inert nitrogen atmosphere to produce monolithic biochar samples. Flat-surfaced pieces were selected and used for hydrophobicity assessment by contact angle measurements. A droplet of deionized water (5 μL) was deposited on the biochar surface using a contact angle measurement device (DMs-401, Japan), and the droplet profile was recorded using side-view optical imaging. This setup enabled precise evaluation of the wettability of the biochar surfaces.

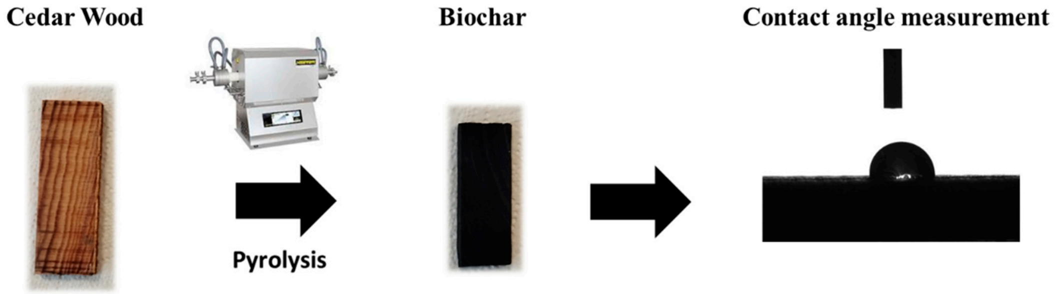

Figure S1. Procedure for contact angle measurement: cedar wood - pyrolysis - biochar monolith - water droplet deposition on flat surface for wettability analysis.

S2. CO_2 adsorption isotherms

CO_2 adsorption isotherms for cedar wood-derived biochar samples pyrolyzed at 800, 900, 1000, and 1100 °C. The isotherms exhibit characteristic of microporous structures. BET surface areas were calculated in the relative pressure range of 0.05–0.30 (P/P_0).

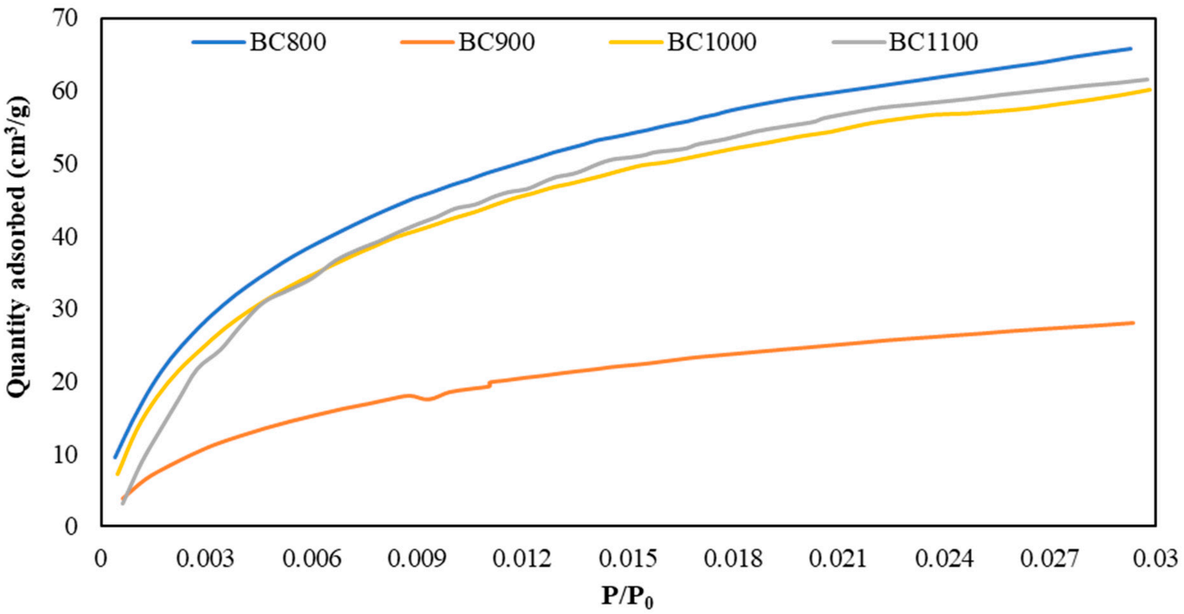

Figure S2. CO_2 adsorption isotherms for cedar wood-derived biochar samples pyrolyzed at 800, 900, 1000, and 1100 °C.

S3. Additional N_2 Adsorption–Desorption Isotherms

To complement the CO_2 adsorption isotherms at 273 K and better understand the porosity evolution of the biochar samples with increasing pyrolysis temperature, N_2 adsorption–desorption measurements were conducted at 77 K on BC800, BC900, BC1000, and BC1100. The resulting isotherms are presented in Figure S3, and the BET surface area values and micropore contributions are summarized in Table S1.

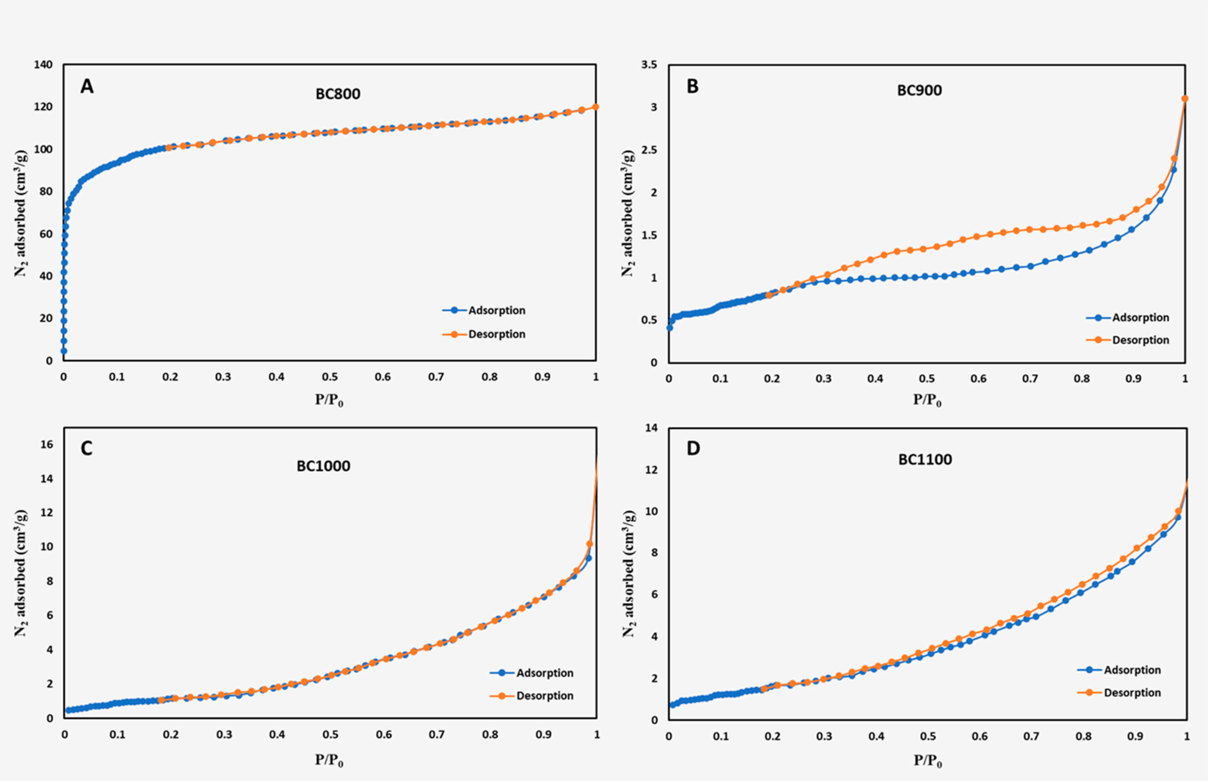

Figure S3. N_2 adsorption–desorption isotherms at 77 K for biochar samples obtained at different pyrolysis temperatures: (A) BC800, (B) BC900, (C) BC1000, and (D) BC1100.

Table S1. Textural properties of biochar samples obtained at different pyrolysis temperatures, determined from nitrogen adsorption–desorption isotherms at 77 K. and from CO_2 adsorption–desorption isotherms at 273 K. SSA: specific surface area; S_{micro}: micropore surface area; S_{meso}: mesopore surface area, V_{micro}: micropore volume; D_{micro}: mesopore diameter.

Material	N_2 adsorption–desorption			CO_2 adsorption–desorption		
	SSA (N_2, m² g⁻¹)	S_{micro} (m² g⁻¹)	S_{meso} (m² g⁻¹)	SSA (CO_2, m² g⁻¹)	V_{micro} (m³ g⁻¹)	D_{micro} (nm)
BC800	370	320	40	454 ± 8	0.024	0.72
BC900	1.7	1	-	385 ± 30	0.161	0.75
BC1000	6.7	-	-	431 ± 40	0.198	0.67
BC1100	7.3	-	-	425 ± 20	0.195	0.73

The data reveal a steep drop in BET surface area from 370 $m^2\ g^{-1}$ for BC800 to values below 1 $m^2\ g^{-1}$ for the higher-temperature samples (BC900–BC1100). This decrease is mainly attributed to the limited diffusion of N_2 molecules into micropores at 77 K, particularly in biochars that become more graphitized and less functionalized at high pyrolysis temperatures. These conditions reduce N_2–surface interactions

and hinder pore accessibility, leading to an underestimation of surface area using N_2. This limitation justifies the use of CO_2 adsorption at 77 K, which offers better access to narrow micropores (<0.7 nm) due to its smaller kinetic diameter and higher quadrupole moment. CO_2 adsorption data thus provide a qualitative indication of residual microporosity in high-temperature biochars, particularly BC900 and BC1000, where N_2-based BET values alone would misleadingly suggest almost no porosity.
